# Genetic Counselors as Research Leaders: A Pragmatic Pathway to Becoming a Federally Funded Principal Investigator in the United States

**DOI:** 10.1002/jgc4.70259

**Published:** 2026-07-17

**Authors:** Heather A. Zierhut, Simone Hetherington, Rachel Mills

**Affiliations:** ^1^ Department of Genetics, Cell Biology, and Genetics University of Minnesota Minneapolis Minnesota USA; ^2^ MS Genetic Counseling Program The University of North Carolina at Greensboro Greensboro North Carolina USA; ^3^ Greenwood Genetics Center Greenville South Carolina USA

**Keywords:** federal funding, genetic counseling, genetic counseling research, grants, National Institutes of Health

## Abstract

In the United States, National Institutes of Health (NIH) federally‐funded research on the practice of genetic counseling is most often led by Principal Investigators (PIs) who are not genetic counselors (GCs). The aim of this study was to better understand the reasons for this and gain insight into how GCs successfully become PIs of NIH federally‐funded research. Using a pragmatic framework, we identified specific skills and supportive pathways that help GCs lead collaborative research. Building on a previously published review of NIH‐funded genetic counseling research, the NIH RePORTER database was used to identify GC and non‐GC participants. Semi‐structured interviews were conducted with GC PIs (*n* = 9) and non‐GC PIs (*n* = 7). Transcripts were analyzed using inductive content analysis and co‐coding. Four overarching categories were identified. 1. Facilitators of GC leadership in NIH‐funded research included: varied research roles, institutional resources, mentorship, GC‐specific skills and traits, supportive teams, assistance with grant review, training opportunities, and requests for applications (RFA) specific to genetic counseling. 2. Identified barriers included institutional restrictions and priorities, limited time and resources, perceptions of Master's credentials, limited familiarity with the grant process, and low confidence. 3. Participants provided guidance for aspiring GC PIs and 4. GCs discussed the process of becoming a GC PI. Both individual and systemic factors were found to impact research leadership development. GCs pursuing research leadership may benefit from mentorship, employment in settings supportive of research, supplemental training (e.g., grant‐writing, research methodology), building a publication record, collaborating with interdisciplinary teams, and adopting a mindset of continual learning. Both GCs and non‐GCs can advocate for institutional and funding policy changes. The incorporation of more GCs as PIs on federal grants can ensure that experts in genetic counseling are leading research that has the potential to influence their practice and the profession as a whole.

## Abstract (highlights)

**What is known about the topic?:**

Genetic counselors are significant contributors to research, holding a variety of research‐related roles, yet few serve as principal investigators leading federally funded grants.

**What this paper adds to the topic?:**

This pragmatic study offers valuable perspectives on key barriers impacting genetic counselors' ability to lead research teams as principal investigators and access federal funding. The results outline strategic, supportive pathways to help genetic counselors develop the necessary skills, obtain necessary resources, and gain critical experiences to become federally funded principal investigators.

## 1 Introduction

Genetic counseling is an essential healthcare service with a growing evidence base demonstrating the value and outcomes related to integration of genetic counselors (GC) into healthcare delivery models. However, a review of awards funded by the National Institutes of Health (NIH) indicates that researchers who are not GCs are leading federally funded research that can impact genetic counseling, with only 9 of these grants (20.9%) awarded to genetic counselors (Zierhut et al. 2024). These studies are often investigating models to replace or supplement genetic counseling and the work of GCs (Zierhut et al. 2024). Concurrently, there are few funded large‐scale empirical studies that investigate the processes GCs use and the outcomes of genetic counseling (Higgs et al. 2023). As such, currently and previously funded research on genetic counseling alternatives is likely occurring at the expense of investigating the processes, outcomes and value of genetic counseling to patients and the healthcare system. GCs leading and developing the evidence base for the practice may help to reduce the harm of conducting research about GCs without including them in that inquiry.

GCs across various degree types and job titles actively pursue and secure grant funding. In 2024, 8% of respondents to the National Society of Genetic Counselors (NSGC) Professional Status Survey (PSS) reported applying for grant support, with 65% of these applicants (*n* = 126) successfully receiving funding and with 32% (*n* = 39) of those receiving federal grants. Among grant recipients, roles included Principal Investigator (PI; *n* = 24), Co‐Principal Investigator (Co‐PI; *n* = 41), and Co‐Investigator (*n* = 48) (National Society of Genetic Counselors 2024). Other genetic counseling‐specific research was funded by private foundations or professional organizations (NSGC 2024). GC involvement in research has been predominantly focused on student‐led research and individual characteristics of GCs such as interest in, motivations, and confidence in the research process (Sikkink et al. 2023). The most common motivator for engaging in research activities was for career advancement, but collaboration with colleagues, students, other institutions, and overall aspects of team building were highlighted as other important factors in their interest. Despite the majority of GCs preferring a more collaborative role, a subset of GCs do have aspirations to develop and lead their own research. Applying for funding was the area of research that GCs were the least confident in (Sikkink et al. 2023).

The underrepresentation of GCs in federal funding points to areas for improvement in support and skills development for genetic counseling research leaders and inclusion of GCs in PI and Co‐PI roles. All GCs trained in ACGC‐accredited programs have some research‐related experiences, but it is unclear how or if an MS degree in genetic counseling prepares GCs to lead collaborative research and achieve awarding of federally‐supported research grants. We aimed to better understand how GCs become PIs on federally‐funded grants and the rationale for the lack of GC researchers leading grants (Zierhut et al. 2024). Our primary research question was “What factors enable GCs to lead collaborative genetic counseling research?” Three sub‐aims investigated (1) What roles GCs have on federally funded research projects about genetic counseling, (2) What skills do GCs need to become PIs for federally funded research, and (3) What facilitators and barriers exist to GCs leading federally‐funded research?

To do so, we assessed perceptions regarding the skills and experiences needed to be awarded a federal grant from PIs who are not GCs as well as GCs that had been either a PI or Co‐I on a federally funded grant. The pragmatic goal of this research was to take insights from those successful in obtaining funding to identify the most common barriers, provide a realistic outlook of the barriers to securing federal funding, and promote supportive pathways for GCs to build the necessary skills and experiences.

## 2 Methods

Researchers engaged in genetic counseling research were interviewed to identify factors that influence GCs to lead collaborative research supported by federal research funding. As the goal of the research was to promote supportive pathways for GCs to build the necessary skills and experiences, we utilized a pragmatic framework (Creswell and Poth 2018). We utilized multiple data sources including publicly available research databases (Zierhut et al. 2024), existing data on research GCs (NSGC 2024), and interviews of genetic counseling researchers including those who are and are not GCs. Research databases and the Professional Status Survey informed the analytic process most importantly by identifying the eligible participants, informing probing questions we asked non‐GC and GC participants, and interpretation of the interview data. The University of North Carolina Greensboro Institutional Review Board and the University of Minnesota designated this research as exempt (IRB‐FY23‐455 and IRB‐22332).

### 2.1 Participants

#### 2.1.1 Non‐GC PIs

Eligible non‐GC participants were PIs of NIH‐funded research studies related to genetic counseling that were identified in a review of NIH RePORTER (Zierhut et al. 2024). Non‐GC PIs were chosen for interviews since prior research has not identified reasons for why they included or declined to include GCs as Co‐Is. Interviewing non‐GCs therefore was an attempt to better understand how/why GCs were/were not involved in research, and non‐GC PIs would be the most appropriate population to answer that question. Including a diverse group of NIH‐funded researchers with variable training experiences and career paths in research also provided diverse perspectives and insight into facilitators, barriers, and practical guidance and allowed for some comparison between the two groups to provide insight into what might be GC‐specific factors. Forty‐two grants led by 36 PIs and Co‐PIs met eligibility criteria (Zierhut et al. 2024). Researchers emailed eligible individuals with an invitation to participate.

#### 2.1.2 GC PIs and Co‐Is

Eligible GC participants were also identified through the previously mentioned review of NIH RePORTER. Of the 42 grants identified, 7 GCs were awardees and eligible to participate. Recognizing that not all federally funded GCs would be identified in the review (Zierhut et al. 2024), additional participants were recruited from a Google List‐serve of GCs that have or are currently pursuing a doctoral degree, the NSGC Research Special Interest Group, a social media post distributed via LinkedIn, and through snowball sampling. An additional 9 GCs who met eligibility criteria (Co‐I or PI of NIH‐Funded research studies related to genetic counseling research) were identified through these methods; we reviewed NIH RePORTER to confirm they had been awarded funding before inviting them to participate and initiating an interview.

#### 2.1.3 Interviews

Those who agreed to participate completed a single semi‐structured interview using interview guides that differed slightly between the non‐GC researchers (Appendix S1) and GC researchers (Appendix S2) due to the variable perspectives of the two groups. The interviews of non‐GC researchers were conducted via Microsoft Teams by the research team (RM & SH) between July and September of 2023 and lasted an average of 33 min (range: 25–38 min). They were asked how they develop their research teams and the role of GCs in their research; targeted probes clarified their perspectives of GCs and the skills needed by GCs or non‐GCs to lead NIH‐funded research. Interviews of GC researchers were conducted with a member of the research team (HZ) via Zoom between July and August 2024. GCs were asked to describe their paths to becoming a PI, facilitators and barriers to obtaining federal funding, and how training and research skills development such as grant writing impacted their work. The average interview time was estimated at 40 min (range: 25–65 min) but exact times are not available due to inadvertent deletion of the timestamps in transcripts. Interviews were automatically transcribed by Microsoft Teams or Zoom and confirmed by the research team.

#### 2.1.4 Data Analysis of Interviews

To focus on the practical applications of the data and address the limited existing research on the topic, we used inductive content analysis (ICA) to analyze the interviews (Vears and Gillam 2022). Following each interview, the interviewer constructed a memo to document elements of the interview that provided clear insight into the research question. These memos were reviewed by the two coders (RM & HZ), who analyzed transcripts of the interviews. Co‐coding was used to create a shared understanding of the data (Coulston et al. 2025).

Data analysis followed the ICA step‐wise process described by Vears and Gillam (2022). Read & Familiarize: Both coders developed the research and were familiar with project aims and relevant literature. We each reviewed the transcripts and memos to familiarize ourselves with the data. First‐round coding: We identified initial categories of data as “big‐picture meaning units,” many of which were aligned with the questions asked during interviews. Second‐round coding: Then each researcher coded a subset of interviews using Lumivero NVivo (version 14) to identify fine‐grained codes, with RM initially coding interviews of non‐GC researchers and HZ coding interviews of GC researchers. Refining: We reviewed and discussed respective codes, coming to a shared understanding of the codes while considering the differing viewpoints of the participant groups. Through this process, we developed initial categories of the codes. Then, we re‐coded all transcripts using the shared codes and categories. Synthesis and interpretation: Categories were further refined through collaboration, and the final categories, sub‐categories and exemplar quotations were identified.

Although counting of codes and categories is common in traditional ICA, counts are not reported here due to use of co‐coding to derive a richer understanding of data rather than to achieve intercoder reliability (Coulston et al. 2025); as such, accurate counts cannot be determined or reported. Because there was a known number of eligible participants and that number was small (36 non‐GC researchers and 16 GC researchers), all invited participants who responded to the invitation to participate were interviewed, and saturation was operationalized on the individual participant level rather than across the dataset (Legard et al. 2003). To achieve this, interviewers used probing questions to deepen the understanding of the participants' perspectives and used a final open‐ended prompt to share any additional thoughts about GCs in research.

#### 2.1.5 Researcher Positionality

HZ is a research GC and her positionality related to this research is shaped by her professional roles as a PI of the Genetic Counseling Processes Result in Outcomes study and co‐lead of The Genetic Counseling Fellowship in ReSearch Training Program. She brings a perspective informed by doing research and training GCs to be research leaders. Her interest in this research is rooted in a sustained commitment to promote GCs' research and their leadership in federally funded projects. SH was a genetic counseling student at the time of data collection and analysis. She has not held any leadership roles for federally funded research. Prior to joining the genetic counseling profession, she was involved with data analysis for an NIH‐funded study unrelated to genetic counseling. SH has a goal for long‐term involvement in research during her genetic counseling career. RM is a career‐long research GC who has been involved in many aspects of grant writing and conducting federally funded research, but has not served as a PI on federally funded research. She is committed to supporting GC researchers and research.

A key methodological consideration was the potential influence of the interviewer's role as a GC when speaking with non‐GC researchers as well as other GCs who are also researchers in the field. The dynamics could potentially inhibit participants from being completely forthcoming regarding perceptions of weaknesses or areas for improvement for GCs in research. To mitigate the potential for bias, the interviewer initiated the discussion by proactively acknowledging known limitations in current GC training related to NIH‐funded research and managing large grants as well as the variability in research‐related training to actively encourage critical dialogue with non‐GCs. Similarly, GC interviewees with known shared identities or previous relationships with the interviewer were actively encouraged to share information even if the interviewer knew parts of the interviewee's history or perspectives related to questions that they were being asked. Furthermore, some interviews with non‐GC researchers were conducted by a GC student not directly involved in NIH‐funded research, aiming to introduce a comparatively more neutral perspective. Interestingly, this interaction also occasionally served as a clarifying point, as non‐GC participants sometimes used the interviewer's expertise to ask clarifying questions about GC training and scope of practice, leading to a richer, bidirectional exchange of professional perspectives.

## 3 Results

Seven of the 36 eligible non‐GC researchers (19.4%) and 9 of the 16 eligible GC researchers (56.2%) completed the interviews. Demographic characteristics were not disclosed to ensure participant anonymity and prevent the risk of deductive identification. We identified 66 NVivo codes in the second round of coding which were refined to create 31 codes organized into four categories. Three categories were present in interviews of both GCs and non‐GCs: *facilitators* that supported the process and experience of receiving federal funding; *barriers* impacting the awarding of federal funding; and *guidance for researchers* who are considering pursuing federal funding. Within these three categories, some codes were present among only one group of participants. The fourth category of codes, *process of becoming a GC PI*, was identified primarily in the interviews of GCs and included codes that described the processes and experiences that promote development of research GCs to become PIs. Some codes fit into multiple categories; for example, interviewees may have described a *facilitator* or *barrier* in a way that was framed as *guidance for researchers* or a factor in the *process of becoming a GC PI*.

### 3.1 Category 1. Facilitators to Genetic Counselors Leading Federally Funded Research

Eight subcategories of facilitators were described by both GCs and non‐GC researchers: (1) Research roles and related responsibilities; (2) Institutional resources; (3) Mentors; (4) GC‐specific skills and traits; (5) Supportive Co‐Is and teams; (6) People to review grants; (7) Training opportunities for writing grants and obtaining research skills; and (8) Request For Applications (RFA) specific to genetic counseling.

#### 3.1.1 Research Roles and Related Responsibilities

GCs reported holding important roles on research teams which facilitate and contribute to their ability to learn related research skills and the development of research‐related competencies as a part of their job. Specific types of work that were beneficial were research project management, collecting and analyzing data, and writing papers or assisting with grants. Some GCs also held clinician roles where they provided counseling for the research study by delivering genetic testing results and providing clinical expertise to other collaborators and researchers.

I've been working—doing all kinds of re‐wearing my other research hats. [An academic mentor] encouraged me to start writing grants and being involved in other types of genetic counseling research. In addition to some of the [grant name], I had on‐the‐job learning. I helped to throw together aims, make figures, and read things for consistency and clarity. (GC003)

I was a project manager. I would make myself useful in asking questions, helping design the research methods, all of that stuff as a part of the grant. Once you're managing it, you know the logistics. The PIs appreciated when I was doing my first grant that I was pulling everything together. (GC009)

Non‐GCs identified many of the same research roles and responsibilities mentioned by GCs, including data collection, organization, and manuscript writing, which ultimately contributed to GCs having a central role in producing rigorous and impactful research that led to publication and additional research opportunities. GCs were noted to have strong genetic and genomic knowledge, enabling them to quickly interpret literature and support grant writing with up‐to‐date scientific understanding. They excelled at translating complex scientific concepts into clear, accessible language—an essential skill for both patient/participant communication and research dissemination. GCs often led teams, managed multi‐year projects, and coordinated across disciplines, demonstrating strong project management and operational leadership which non‐GCs acknowledge is necessary for leading NIH grants. Their positions were often integrated into grant proposals, and they contributed to developing interventions and refining study materials during early research phases.

We couldn't do without them. They need to collect all the data, they need to organize it. They even write the papers. (non‐GC100)

Their expertise is key for this type of research. They are the experts on all the, not only on the content, but also they have a lot of expertise in how to communicate about cancer risk. (non‐GC102)

One of the things that [GC] was particularly amazing at, and the reason that she brought so much value to the grant was because she knew how to lead a team, and she was in charge of leading our full genetic counseling team, and she understood the ins and outs of operations. (non‐GC103)

#### 3.1.2 Institutional Resources

GCs and non‐GCs considered access to resources and institutional support to be a critical component to successful submission of federal grants that led to funding. Supervisors, department heads, and those in administrative positions were helpful in supporting the GCs' awareness of and connection to resources. In some cases, these were direct outreach to potential collaborators or financial resources such as start‐up funds or institutional grants. Grant specialists and the grant office that helped collate the pieces of the grant including budgeting were particularly helpful. GCs often needed these specialists and offices to submit grant progress reports (project advancements made during the budget period), financial reports (funds spent according to the approved budget and federal cost principles), and compliance and other administrative reports (risk and legal documentation).

So that [grant specialist] is a resource that is extremely helpful. You need [one]. If I didn't have that person, I'm not even sure I'd continue doing grants. (GC001)

When we get ready to apply for a grant we meet with our pre‐award people. They help us go through the checklist. We give them all the information, they upload it. (GC005)

Online training or institution‐specific training or things like that could also kind of give a more research intensive, you know, exposure. (non‐GC105)

#### 3.1.3 Mentors

Mentorship was noted by all of the GC PIs and non‐GCs as a critical part of their success. GCs described receiving encouragement from mentors to pursue research activities, including grant writing and collaborative leadership roles such as Co‐PI or Co‐Is. Access to experienced, NIH‐funded investigators further enhanced research capacity by offering structured mentorship grounded in successful funding and publication experience. Mentors provided technical and emotional support but also strategic guidance, helping GCs articulate personal research missions and develop long‐term research agendas.

[My mentor is] very good at strategic planning. Together, we did a lot of meetings to develop my personal mission. And what my research agenda could look like, and how I can get there. (GC007)

Then I had excellent research mentorship at [location and name of mentors]. These were all people who had a past history of being successful. Either NIH PIs or on NIH Funded Grants. So I had a lot of successful NIH funded investigators around me to mentor me and help. (GC008)

Non‐GC researchers recommended that GCs work with experienced investigators who can provide structured guidance. Senior GCs with substantial research experience were also recognized as potential mentors. Effective mentorship models often involved interdisciplinary collaboration, such as GC–physician co‐investigator pairings (e.g., with oncologists). Collaborations with well funded researchers were perceived as particularly successful. Mentorship typically followed a model, wherein they guided early‐career GCs through project development, study design, research execution, and publication, fostering research skill acquisition at each step through hands‐on experience.

I tend to work very closely with [GCs], to kind of help mentor, and, and help them do, and then they totally understand it. But they don't walk in knowing it. (non‐GC105)

#### 3.1.4 GC‐Specific Skills and Traits

GCs brought counseling skills and clinical expertise to study development and helped non‐clinical oriented team members understand what was feasible or not. GCs described leveraging a diverse set of transferable skills. Skills developed during their fieldwork training and clinical positions were seen as directly applicable to research activities such as coordination, organization, time management, and multitasking. Participants also emphasized the development of scientific writing and grant preparation skills that were gained often through iterative practice and mentorship. GCs highlighted their collaborative tendencies, openness to interdisciplinary teamwork, and ability to contribute clinical insights into the patient experiences and clinical appropriateness of research protocols. Additionally, some GCs brought specialized methodological expertise, further enhancing their value to research teams.

We have such valuable skills. There is so much focus on…how to deliver our services, how to meet this need…be open and be willing to be part of those teams. You will have a lot to contribute. (GC005)

I think that patient interaction [is important], being a GC, just watching and observing patients and understanding how they're viewing things. (GC004)

Non‐GC researchers affirmed that GCs bring a diverse and adaptable skill set that would be valuable in contributing to and leading research teams. Several respondents noted that GCs possess strong communication skills, enabling them to serve as effective scientific communicators across audiences, including in writing, presentations, and patient and participant‐facing materials. GCs were also described as highly adaptable and team‐oriented, often stepping into complex roles and contributing to service delivery design and team building. Their clinical training prepared them for the scientific rigor and psychosocial insights of research, which allowed them to navigate nuanced discussions and maintain a clear orientation toward patient‐centered outcomes. Researchers also highlighted that personal attributes such as work ethic and intrinsic motivation further distinguished GCs as valuable contributors to their interdisciplinary research teams. A few researchers noted, however, that motivation and interest in pursuing roles such as PI varied by each GC.

[My colleague] always likens our GCs to navy seals because they can jump in and do everything…They really are so good at just jumping in and doing what needs to be done and figuring things out, which I think is a really valuable skill set. (non‐GC105)

[The GC's] contribution had everything to do with the service delivery and the quality of that service, how to build that team, how to do it in this really efficient way. (non‐GC103)

GCs have a natural orientation, like a trained orientation that's really helpful. In my experience, I have found that GCs are extremely outcome oriented and are never the people to forget why you're doing something. Like a very good forest for the trees, sort of thinking. (non‐GC106)

#### 3.1.5 Supportive co‐Investigators (Co‐Is) and Teams

GCs emphasized the importance of intentional inclusion and collaboration of Co‐Is in supporting GCs' advancement in research; non‐GCs expressed similar sentiments. Strategic team‐building helped to create a strong, multidisciplinary team comprised of a group of researchers that have the necessary expertise that is needed to carry out the research. Identifying topic‐specific expertise and accessing professional networks was a common approach to assembling research teams. Effective collaboration was described as foundational to research success, with participants noting that the ability to work effectively within interdisciplinary teams is critical for all PIs.

Non‐GC participants highlighted the value of involving GCs throughout the research process, including assigning writing responsibilities and offering primary (first) authorship as a way to be a supportive team member. This approach was seen as instrumental in building GCs' confidence, increasing their exposure to research processes, and supporting the development of independent research skills over time. Several Non‐GC respondents described inviting GCs to serve as Co‐Is, particularly in studies focused on genetic counseling practice, where their domain‐specific expertise was considered indispensable.

I think you need a team no matter what. Even experienced researchers need a team. (GC001)

Other academic PIs that I work with that are within [specialty area], have been the biggest encouragement in trying to build my research career. (GC003)

There is nothing that happens without deep and extensive collaboration, so if you cannot collaborate with people you will not be successful as a PI. (non‐GC106)

#### 3.1.6 People to Review Grant

GCs pursuing grant funding valued detailed expert feedback throughout the proposal development process. Engaging with colleagues who were willing to contribute substantively to writing and problem‐solving was described as highly beneficial. In particular, early involvement of external collaborators as well as informal reviewers was instrumental in refining proposals. Feedback on specific components, such as the specific aims page, was impactful when provided by individuals with insight into funder expectations to elevate the clarity, focus, and competitiveness of grant submissions.

Non‐GCs noted that formal training in grant writing was lacking among GCs; however, it was not unique to GCs as they had similar experiences in their own professional education. They underscored the universal need for mentorship and peer support to build these competencies. Actively writing grants and receiving iterative feedback were noted as a means for assisting GCs with skill acquisition. Hands‐on experience and reviewing successful model grant applications were considered particularly helpful. Several non‐GCs described serving on study sections to help their understanding of funder expectations.

I heard that a particular person was very, very good at reviewing specific aims pages. So I sent it to her, and I mean she just made it so much more strategic, higher level. She knew what they would want to see, so that was really helpful. (GC008)

I got lucky to be around other people who write good grants and to read their grants, and eventually that led to me writing grants, and eventually that led to me, being on study sections, where you get to look at a lot of grants with a very critical eye. (non‐GC101)

#### 3.1.7 Training Opportunities for Writing Grants and Obtaining Research Skills

Training opportunities for grant writing and research skill development during masters training were highly variable. Some GCs felt very prepared after their genetic counseling graduate program and others did not. In general GCs' programs provided some helpful preparation in addition to the clinical expertise that they gained from the genetic counseling degree. Most GC and non‐GC interviewees thought that additional training related to research methodologies and grant writing was needed. Educational opportunities beyond the MS alone would have been helpful. A few researchers noted the challenges to lead a federal grant or be a PI even with a doctoral degree and post‐doctoral training.

Graduate training for me did not prepare me at all to be a PI. Now granted, this was a very long time ago. (GC006)

I don't think a new grad should or could do a good job. I don't even think a new grad from a PhD. I think they need more experience before they can truly be in charge. (GC001)

The focus of most of the training recommendations was related to study design and generally how to generate a set of methodologies to test a research question. Some GC researchers noted that they were continually learning and needed to do so as a part of the research process for the job. Grant writing workshops, fellowships, and other programs that support research skills development were pursued. Categories of trainings that GCs found helpful included: (1) grant writing; (2) qualitative research methodology; (3) statistics; (4) implementation science; (5) basic science research; and (6) other methodology training. Several specific trainings were cited, including NSGC workshops and NIH‐sponsored implementation programs. Non‐GCs also suggested educational opportunities related to implementation science, statistics, qualitative research training, and grant writing. They also suggested informal or experiential learning activities like journal clubs, manuscript review, and motivational interviewing training.

Non‐GCs recognized the importance of formal research training mechanisms. Postdoctoral training was described as an effective pathway for skill development such as NIH‐funded T32 training grants or K awards. These funded experiences were valuable entry points for pursuing research careers and instrumental in helping them build a track record of independent research while receiving structured mentorship.

#### 3.1.8 Request for Applications (RFA) Specific to Genetic Counseling

A targeted funding opportunity issued by the National Human Genome Research Institute (NHGRI) focused specifically on genetic counseling research was the mechanism used by several of the GCs. It was noted that this request for applications was the only place where the scope and aims of GC‐led projects were considered a strong fit.

The fact that it was specifically a RFA for genetic counseling processes and outcomes…I would have been pretty hesitant to try to sell this idea exactly as written in another setting. (GC002)

[NIH] really wanted counseling oriented focus grants…I was like, ‘all right, this is it’, [I] came back from that meeting really empowered. (GC004)

One non‐GC observed that within the body of genetic counseling research there were two predominant areas of study: one that examined the practice of genetic counseling itself and the other studied outcomes‐based research evaluating the impact of genetic counseling on patient or system‐level metrics. The former was perceived as more prevalent, while the latter, though less commonly funded, was viewed as critical for demonstrating the long‐term value and effectiveness of genetic counseling interventions.

### 3.2 Category 2. Barriers to Genetic Counselors Leading Federally Funded Research

#### 3.2.1 Institutional Restrictions on GCs Submitting Grants

Although eligible to apply for NIH grants, GCs faced challenges in securing institution‐specific funding to support pilot studies that would build toward a larger NIH proposal. For some GCs, institutional constraints required that they rely on collaborators to submit grants on their behalf. In these cases, formal recognition as a Co‐I was contingent upon securing the grant funding and obtaining dedicated time to meet their institutions' eligibility requirements. Several GCs' pursuit of faculty status was met with significant institutional resistance, requiring a lengthy and labor‐intensive advocacy process. Additionally, GCs with master's degrees encountered barriers to applying for NIH K awards. Non‐GCs noted that granting institutions often prioritize applicants with PhD or MD credentials, creating structural barriers for GCs.

I know at some institutions, they won't even let a GC be a Co‐I, which is ridiculous. But they'll let an MD who usually has less training in research than the GC. (GC001)

I couldn't apply for internal pilot funding. I had to have a partner who would have a MD/PhD. Some of those things were barriers. (GC005)

I'm not eligible for K awards through the NIH, because I don't have a doctoral degree which is limiting. (GC003)

#### 3.2.2 Limited Resources to Assist With Research or Apply for Grants

Some GCs worked in hospitals or non‐academic medical centers that lacked the administrative infrastructure and support systems to submit applications. In a few cases, GCs moved jobs to gain the necessary support to apply for grants. Non‐GCs also noted that resources like personnel were needed to develop grants and build research teams and predicted that GCs would struggle to be successful without these resources. They described a competitive environment with low success rates and believed it would be challenging to be a PI without clear institutional recognition or rewards for pursuing research in leadership roles.

[Leadership said] ‘Yes, we want you to [write grants]. You can apply for grants. But we're not going to give you any resources to apply for grants.’ So it got really frustrating. (GC009)

You need to be able to buy out time or resources for stuff [which GCs do not have]. (non‐GC104)

#### 3.2.3 Limited Time

Though some GCs were encouraged to apply for grants by their institutions, they were also expected to maintain their full‐time workload, leaving little time or flexibility for grant writing. A lack of protected research time was a common barrier among GCs. GCs often developed grants and manuscripts during their personal time. If funded, grants often only covered their role in executing the research, and there was no dedicated research time allotted to developing future proposals or sustaining research engagement. The limited lack of time was similarly recognized as a significant barrier by non‐GCs.

What I would have needed to really transition is like stopping being a good GC like I stopped seeing patients. It was a time thing. (GC005)

GCs don't generate a lot of revenues, so it's a practical problem of how to fund the GC to take the time to give the effort to be a PI on a grant. (non‐GC101)

#### 3.2.4 Negative or Unclear Perceptions of GC Credentials in Research

GCs identified professional and perceptual barriers related to their educational background and holding a master's degree. GCs noted that they were seen as less competitive compared to other researchers with MD or PhD credentials, and this was reiterated by the Non‐GCs. Some GCs and Non‐GCs shared a belief that grant reviewers misunderstood the value of genetic counseling and thus prioritized research awards on uptake of *genetic testing* over research awards on *genetic counseling*. Interviewees perceived grant reviewers as undervaluing or misunderstanding the contributions and significance of GCs in research. Non‐GCs noted that persistent biases and misconceptions hindered GCs' participation in research, and that reviewers in study sections would dismiss applications from those with MS‐level credentials. Reviewers sometimes narrowly defined the GC in their clinical role rather than recognizing their value as researchers within the discipline who are capable of contributing to research. The lack of recognition created a higher burden of proof for GCs to establish their legitimacy as researchers to secure funding.

But even being on study sections at times, they're like ‘you just have your Master's degree, What are you doing here?’ (GC004)

People think of genetic counseling as a clinical position, and as a degree is thought of as a clinical license rather than a research degree. (non‐GC103)

Most grant study sections would probably not take them [GCs] very seriously…Just bias, stereotypes. You have to have a PhD or an MD to get a grant. (non‐GC101)

There are a number of very clear examples where for whatever historical reasons, there is more of a burden to prove in genetic counseling than in other credentials. I'm definitely not dismissing that as a real factor, just trying to be very pragmatic about what are the things that are required for change. (non‐GC106)

#### 3.2.5 Limited Understanding of Grant Process

GCs reported feeling new to the research process and not having been prepared for all the different aspects of the grant process.

Probably the biggest obstacle is that no one ever really taught me how to do it. I kinda teach myself. (GC004)

You wanna make sure that you're doing it right. It's a big thing. So there were no manuals that we could really find. We just tried to ask questions as much as possible. (GC006)

#### 3.2.6 Institutional Funding Priorities That Are Not Focused on Genetic Counseling

GCs and Non‐GCs both reported that funders have not been interested in genetic counseling related research or there is a need to try to masquerade as other types of research to try to get funded for related work.

The current funding mechanism, the environment, it has become more competitive to receive funding, especially niche funding. (GC007)

If you want to go after an NIH award, remember, the thing you have to care about is the research outcome, not the clinical outcome. If you write your awards to focus on clinical outcomes, NIH will see it as not a grant that they should fund. (non‐GC103)

#### 3.2.7 Low Confidence

A lack of confidence in research capabilities and the feeling of imposter phenomenon was raised by a couple of GCs. Non‐GCs expressed uncertainty about the qualifications of GCs and highlighted that structured research training could build confidence and competence in study design.

First, training to be a PI, it's something I still feel like can I do this? Am I doing it right? (GC003)

### 3.3 Category 3. Guidance for Researchers Who Are Considering Pursuing Federal Funding

Consistent with the study's pragmatism paradigm, we identified a collection of codes that constituted practical guidance for others who may consider pursuing federal funding. These recommendations came from GC and non‐GC researchers and are summarized in Box 1. We aimed to capture the tone as well as the explicit and latent meaning of their advice in their guidance.

BOX 1 **Guidance for Forging a Career in GC Research Leadership.**

For all researchers:

- Remind yourself that getting funding and doing research is fun, rewarding, valuable, and makes a difference.
- Writing skills for grant writing are distinct from other types of writing, and these skills are critical to develop, especially if you were not able to do so in your graduate studies
- Reach out to other researchers, plant the seed, and advocate to be involved and eventually lead research.
- Gain experiences necessary to be a successful PI by being part of a larger team.
- Understand the broader research ecosystem and be strategic about the grants you submit.
- Be open to different types of grants and keep an eye out for other types of funding sources.
- Persistence is key! Keep going and you can do it.

For GC researchers:

- GCs' skills are highly transferable, and that includes applications in writing and research.
- GCs are invested in our profession and our clients, so we should be the ones to create an evidence base for the field—leading grants can accomplish this!

### 3.4 Category 4. Process of Becoming a GC PI

GC researchers were asked about their process to becoming a GC PI, including experiences that were integral to their success. Though the paths that led to applying for and being awarded NIH funding differed among participants, they shared many common experiences or factors that propelled them along the way.

The majority of participants valued *grant writing experience* and described working in all aspects of the grant writing and submission process prior to developing their own grant. Although other experiences were important, grant writing experience seemed to be the decisive factor for success in achieving future funding. Some supported grant writing or submission for a collaborator, but not the entirety of the process. Others gained grant writing experience when applying for smaller grants. GC007 said, “Gradually I built my expertise, built one aspect of the research program agenda, and I was able to take the lead on smaller grant applications and then pursue NIH funding as a PI.” Two GCs highlighted the *need to develop grants on the side*, with participant GC006 stating, “Whether that's in addition to your clinical work that might take some of your clinical work time. It's kind of all unofficial that you're doing this work until you get the grant”.

GCs gained on‐the‐job experience that supported their development by *working in research settings*. Many worked in academic medical centers with other researchers or on large consortiums. These roles provided experiences like *grant writing* as noted by participant GC003: “I had been helping write the consortium grants…in addition to some other administrative supplements, so I had on the job learning.” Participant GC009 said “I started working in research after I got my genetic counseling degree and [was] just constantly asking questions.”

Research experiences allowed GCs to create a *long publication and research record* which established them as productive researchers. A strong publication record and set of research projects leading up to the applications for the federal grants gave GCs “that track record of building things” (GC005). Many applied for and received smaller grants that were internal to their institution or were GC‐specific grants like NSGC's Jane Engelberg Memorial Fellowship (JEMF). These small grants helped to build their record and succession of a productive research record for obtaining a larger grant as PI.

*Interdisciplinary teamwork* also prepared GCs to lead research as PIs. Interviewees described collaborating with teams of other GCs and other researchers. Interdisciplinary work enabled them to craft teams based upon different skills and areas of expertise to be complementary to their own skillset. Participant GC004 noted the complementary nature of their teams saying “We became a really good kind of tag team. [My collaborator] knows nothing about genetics. I know nothing about behavior science.” Interdisciplinary collaboration also led to learning and mentorship opportunities, as described by GC006: “A couple of GCs had been part of a grant writing process, though with other PIs, who kind of had an insight into what the process looked like and wanted to take it a step further for their own learning.”

This *continual learning* was also referenced as an important experience and frame of mind along the path to becoming a PI. As a GC researcher, GC003 described themselves as “a constant learner, and you're sort of set up to always be [a learner].” Even experienced GC PIs acknowledge their continuous learning, with GC001 stating, “You definitely learn as you go. I'm continually learning things. I taught myself the methodology because nobody at my institution knew anything about it.” Though many GCs expressed that they learned on the job and that the process was one of continual learning of new skills and methodologies, two noted the value of formal education, specifically *doctoral training*. GC009 stated that they “fully intended to do this all with my Master's degree” but pursued a doctorate because of an opportunity to do so at their place of work, acknowledging, “I knew that was going to help with what I wanted to do.”

## 4 Discussion

Interviews of NIH‐funded investigators, including both GC and non‐GC researchers, revealed factors that influence the emergence of pathways for GCs as leaders in research. Determinants were present at the individual and systematic level, encompassing elements common to the broader research community and those specific to GCs. Our study employed a pragmatic approach strengthened by implementation of data triangulation through incorporating multiple evidence streams. Data included an analysis of previously reviewed federal grants (Zierhut et al. 2024), complemented by in‐depth, semi‐structured interviews with both federally funded GC and non‐GC researchers. The combination of perspectives provided a depth of understanding related to the interconnected factors, facilitators, and structural barriers influencing research leadership development.

The pragmatic approach enabled identification of actionable strategies that GCs can use to help promote success in submitting and receiving federal funding as a PI. The process of becoming a PI involved many different pathways with variable training and support, and we aimed to provide a holistic view and concrete guidance for GC researchers on those paths. Early career decisions influenced long‐term trajectory and some GCs needed to take additional steps compared to non‐GCs. Some important individual‐level considerations that GCs can take when navigating important decisions include: finding mentors, choosing a workplace that will support research aspirations, supplementing research methodological training, prioritizing grant writing skills, establishing a research agenda and creating an associated publication record, and advocating for systematic‐level changes.

### 4.1 Finding Mentors

Genetic counseling experts have acknowledged the importance of mentorship to their professional development (Miranda et al. 2016), and mentors were critical to becoming a PI among our participants. GCs can cultivate a supportive network comprised of mentors with distinct and sometimes overlapping roles. This involves intentionally seeking mentors with varied expertise, including content experts to guide research focus, methodology experts to ensure rigorous study design, and experienced grant writers to refine proposals. Our findings and those from nationwide programs such as National Research Mentoring Network support engagement in formal mentorship programs, like those offered by the NSGC, increasing the success rate for federal funding (Weber‐Main et al. 2020). They also simultaneously build informal, long‐term collaborations that can evolve into sustained mentorships (Weber‐Main et al. 2020). GCs should prioritize gaining practical research experience with the oversight of more experienced researchers and formal programs that coach them on grant‐writing skills. This means serving as a junior researcher or Co‐I under the guidance of an established PI to acquire hands‐on experience and demonstrate the capacity to successfully contribute to a large‐scale research project. By combining an approach to mentorship that creates a broad strategy with direct, hands‐on research roles, GCs aiming to lead grants can build the foundational skills, professional credibility, and support network necessary to succeed as independent investigators.

### 4.2 Choosing a Workplace That Will Support Research Aspirations

Interviews with GCs and non‐GCs suggested that the workplace environment and available resources are critical for fostering researchers' development. Researchers broadly describe grant development support, research administration, and resources from the University as top factors in their importance of facilitating or hindering their success on grants (Goff‐Albritton et al. 2022). Therefore, to support the development as research leaders, GCs may consider strategically choosing to work at an institution that supports their involvement in research‐related roles. A supportive research environment with existing research infrastructure and expert support provides a foundation of practical resources for researchers. Key resources included: a grants office or research administration team to help with proposal development, budgeting, and submission; access to free or low‐cost continuing education opportunities to develop skills in research methodology and grant writing; and additional resources to conduct research such as a biostatistics core, laboratory support, and a network of potential collaborators.

Beyond these tangible resources, the workplace culture and policies must also be conducive to GC researchers (Goff‐Albritton et al. 2022). GCs who wish to become research leaders should choose employment at institutions that do not limit faculty and PI status to those with a doctorate since faculty status may directly impact eligibility for funding. GCs should also advocate for protected time for research within their roles, acknowledging that a balance between clinical duties and research is crucial for success.

### 4.3 Supplementing Research Methodological Training

Genetic counseling training programs provide essential foundational knowledge for genetic counseling practice; however, the research‐related training that is typical of a MS degree was not enough to prepare a GC to lead a large‐scale research project. The skills learned in these programs are highly complementary and provide a unique advantage, such as strong time management and deep clinical expertise; however, to bridge the gap between foundational clinical genetics training and the demands of a PI role, it was recommended that GCs supplement with methodological research skills training. Of note, doctoral training is not a prerequisite for success, but for some people a PhD is the most efficient and effective way to gain methodological training and skills. For those who want to take a different research path to becoming a PI, specialized fellowships that provide immersive research experience may be another effective way to gain supplemental training (MacFarlane and Zierhut 2024). Continuing education, including workshops and courses, can also be used to continually update expertise in research methods and grant writing. Continuing education is a requirement for genetic counseling recertification (American Board of Genetic Counseling (ABGC), n.d.). Less formal methodological training may also be beneficial and include actively engaging in doing research under the guidance of an expert trained in that specific methodology. Adapting a research skillset over the course of a researcher's career is an ongoing process of learning and collaboration. These strategies can support PIs in gaining the necessary methodological background to develop and execute high‐impact, innovative and rigorous research studies.

### 4.4 Prioritizing Grant Writing Skills

Securing funding is a critical component of a successful research career, but is often under‐developed among new research leaders (Baker et al. 2025). To secure funding from any awarding institution, including federally‐funded institutes like the NIH, a GC must develop strong grant‐writing skills. Grant writing skills development is a continuous, iterative journey of learning and practice. GCs can begin this learning process by targeting small grants from foundations or professional organizations, such as the Audrey Heimler Special Projects Award or the Jane Engelberg Memorial Fellowship offered through NSGC. These grants provide a valuable opportunity to practice the grant‐writing process on a smaller scale, building confidence and a track record of funding success. Smaller grants offered through universities and private institutions or advocacy groups can also be good entry points for funding. As a researcher's skills and experience grow, they can progressively work toward applying for larger, more competitive grants from larger agencies like the NIH. To further develop grant writing skills, GCs can gain hands‐on experience by writing sections of other PIs' grants as a Co‐I or research team member. This provides a direct, low‐stakes way to learn the structure, tone, and key components of successful proposals.

Additionally, GCs can pursue formal training in grant writing and work closely with mentors and a review team to receive constructive feedback and iterate on drafts (Wisdom et al. 2015). Mentorship and review can be particularly important for developing a budget for grants as this is often a new part of the process and not something that is easily known or transferred from other types of writing. Mentorship and institutional support can also provide examples of well‐written grant templates and exemplar grants to guide future applications. Over time and through practicing the skill, participating in formal training, and learning strategies and technical aspects of the grant‐writing process, GC researchers gradually build their research programs by applying for and securing larger grants.

### 4.5 Establishing a Research Agenda and Creating an Associated Publication Record

Developing a successful research career requires a deliberate and strategic approach, moving beyond a series of disconnected projects. It's crucial for an aspiring GC researcher to establish a coherent research agenda that guides their work and creates a focused publication record. This is an active process that begins with a clear mission or vision. To achieve this, GCs can first outline their long‐term goals and aspirations. This involves identifying a set of high‐level research questions that are both personally meaningful and relevant to the field. Their research work will then be strategically focused on these questions, ensuring that each new project builds on the last. This approach, where each study is a building block in a larger framework, is essential for demonstrating expertise and building a compelling record of publications that highlights a clear and impactful contribution to the field. While this agenda and the specifics can and will evolve over time, this intentional, non‐random process is the foundation for a thoughtful, cohesive, and influential research career.

### 4.6 Advocating for Systematic Changes

The practical guidance described thus far has focused on actions of the individual researcher and specifically NIH funded research in the United States; however, advancing the research capabilities of GCs requires advocating for broader, systemic changes within academic institutions and a variety of funding bodies worldwide. For instance the NIH K‐awards policy explicitly states in their eligibility criteria that candidates must hold a research or professional doctoral degree (e.g., PhD, MD, PharmD). Because the standard entry‐level degree for genetic counselors is a MS, GCs are structurally excluded from these foundational “on‐ramp” grants for independent research. Additionally barriers to obtaining funding as reviewed in our research may mirror the systemic barriers to disseminating research findings, demonstrating challenges for GCs in multiple phases of grant cycle (Blanche et al. 2024). Additionally, while female‐dominated professions are systematically undervalued in research resource allocation, regardless of PhD status, this point did not come up in our interviews. However it is possible that these multiple intersections of identity in genetic counseling may be additive and related to the field being primarily composed of women (Ruediger et al. 2025).

Advocacy work is common among GCs as we champion the profession and promote policies that improve access to care and patient outcomes (Caffrey 2022). The researchers in this study recognized that many of the barriers to a successful research career are institutional and can only be overcome through proactive advocacy by GCs and non‐GCs. By actively working together to create a more supportive ecosystem for aspiring researchers, the field of genetic counseling research will improve and create meaningful changes. The advocacy efforts fall into three key areas. First, GCs can advocate for changes in institutional and departmental policies that prevent GCs from being appointed as faculty, serving as a PI, or being eligible for certain funding mechanisms. GCs provide a powerful voice to internally address these inequities in access to grants within their places of employment and can leverage their professional networks and academic partnerships. Second, GCs can advocate for actively creating and supporting research training opportunities specifically tailored to the needs of our profession, such as specialized workshops, fellowships, and continuing education courses that can boost overall readiness to participate in research and skills development (MacFarlane and Zierhut 2024). Finally, GCs can “pay it forward” by providing dedicated mentorship to junior researchers, particularly focusing on grant writing. These advocacy efforts will help build the next generation of GC researchers and ensure a sustainable pathway of talent, reinforcing the value of our profession as a source of innovative and impactful research.

## 5 Conclusion

In summary, we outlined a pragmatic pathway for GCs aspiring to lead federally funded research using insight shared by successfully funded PIs. By focusing on real‐world challenges and successes in NIH funding, the study yielded a crucial set of recommendations designed to bridge the gap between GCs' clinical‐focused MS training and research leadership. The results offer concrete guidance aimed at overcoming common individual and systemic barriers that GCs often face in the grant submission process in the United States. Ultimately, the successful integration of GCs as PIs on federal grants is essential for significantly advancing the scientific rigor and impact of genetic counseling research.

## Author Contributions

Heather A. Zierhut: Concept and design; recruitment; data collection; data analysis and interpretation; writing – initial draft and editing. Simone Hetherington: Data collection; writing – initial draft and editing. Rachel Mills: Concept and design; recruitment; data collection; data analysis and interpretation; writing – initial draft and editing; supervision.

## Funding

This work was supported by the University of Minnesota.

## Ethics Statement

Human studies and informed consent: This study was approved by the University of North Carolina Greensboro Institutional Review Board (IRB‐FY23‐455) and the University of Minnesota (IRB‐22332). Participants provided informed consent prior to participating in interviews.

Animal studies: No non‐human animal studies were carried out by the authors of this article.

## Conflicts of Interest

The authors declare no conflicts of interest.

## Supporting information


**Appendix S1:** Interview Guide for Non‐GC Researchers.

**Appendix S2:** Interview Guide for GC Researchers.

## Data Availability

The data that support the findings of this study are available on request from the corresponding author. The data are not publicly available due to privacy or ethical restrictions.

## References

[jgc470259-bib-0002] American Board of Genetic Counseling (ABGC) . n.d. “How to Recertify.” https://www.abgc.net/Recertify/How‐to‐Recertify.

[jgc470259-bib-0003] Baker, V. L. , S. Starck , and M. Rising . 2025. “Required, but Not Developed: Academic and Grant‐Writing Skill Development on the Path to the Professoriate.” Trends in Higher Education 4, no. 1: 14. 10.3390/higheredu4010014.

[jgc470259-bib-0004] Blanche, E. , T. Wainstein , A. Dey , GenCOUNSEL Study , and A. M. Elliott . 2024. “Genetic Counselors' Research Dissemination Practices and Attitudes.” Journal of Genetic Counseling 33, no. 2: 413–424. 10.1002/jgc4.1743. 37382025

[jgc470259-bib-0005] Caffrey, R. G. 2022. “Advocating for Equitable Management of Hereditary Cancer Syndromes.” Journal of Genetic Counseling 31, no. 3: 584–589. 10.1002/jgc4.1548. 35032082

[jgc470259-bib-0006] Coulston, F. , F. Lynch , and D. F. Vears . 2025. “Collaborative Coding in Inductive Content Analysis: Why, When, and How to Do It.” Journal of Genetic Counseling 34, no. 3: e70030. 10.1002/jgc4.70030. 40305144 PMC12042989

[jgc470259-bib-0007] Creswell, J. W. , and C. N. Poth . 2018. Qualitative Inquiry & Research Design: Choosing Among Five Approaches. 4th ed. SAGE Publications.

[jgc470259-bib-0008] Goff‐Albritton, R. A. , P. A. Cola , J. Walker , J. Pierre , S. D. Yerra , and I. Garcia . 2022. “Faculty Views on the Barriers and Facilitators to Grant Activities in the USA: A Systematic Literature Review.” Journal of Research Administration 53, no. 2: 14–39.

[jgc470259-bib-0009] Higgs, E. , K. E. Wain , J. Wynn , et al. 2023. “Measuring Quality and Value in Genetic Counseling: The Current Landscape and Future Directions.” Journal of Genetic Counseling 32, no. 2: 315–324. 10.1002/jgc4.1657. 36385723 PMC12207684

[jgc470259-bib-0019] Legard, R. , J. Keegan , and K. Ward . 2003. “In‐Depth Interviews.” In Qualitative Research Practice: A Guide for Social Science Students and Researchers, edited by J. Ritchie and J. Lewis , 139–169. Sage.

[jgc470259-bib-0010] MacFarlane, I. M. , and H. Zierhut . 2024. “Promoting the Integration of Genetic Counseling Education and Research Across the Spectrum of Learners at a Large Academic Institution.” Journal of Genetic Counseling 33, no. 1: 250–254. 10.1002/jgc4.1810. 37864570 PMC10923058

[jgc470259-bib-0011] Miranda, C. , P. M. Veach , M. A. Martyr , and B. S. LeRoy . 2016. “Portrait of the Master Genetic Counselor Clinician: A Qualitative Investigation of Expertise in Genetic Counseling.” Journal of Genetic Counseling 25, no. 4: 767–785. 10.1007/s10897-015-9863-3. 26275666

[jgc470259-bib-0012] National Society of Genetic Counselors . 2024. “Professional Status Survey.” https://www.nsgc.org/Policy‐Research‐and‐Publications/Professional‐Status‐Survey.

[jgc470259-bib-0013] Ruediger, S. , L. Ris , and K. Iyer . 2025. “Equitable Research Funding: Strategies, Challenges and the Role of Funding Agencies.” European Journal of Neuroscience 61, no. 11: e70160. 10.1111/ejn.70160. 40495753

[jgc470259-bib-0014] Sikkink, K. , P. Delk , L. Wetherill , A. Breman , and M. Wesson . 2023. “Factors That Influence Genetic Counselors' Participation in Research.” Journal of Genetic Counseling 32, no. 2: 351–361. 10.1002/jgc4.1642. 36210790

[jgc470259-bib-0015] Vears, D. F. , and L. Gillam . 2022. “Inductive Content Analysis: A Guide for Beginning Qualitative Researchers.” Focus on Health Professional Education: A Multi‐Professional Journal 23: 111–127. 10.11157/fohpe.v23i1.544.

[jgc470259-bib-0016] Weber‐Main, A. M. , R. McGee , K. Eide Boman , et al. 2020. “Grant Application Outcomes for Biomedical Researchers Who Participated in the National Research Mentoring Network's Grant Writing Coaching Programs.” PLoS One 15, no. 11: e0241851. 10.1371/journal.pone.0241851. 33166315 PMC7652313

[jgc470259-bib-0017] Wisdom, J. P. , H. Riley , and N. Myers . 2015. “Recommendations for Writing Successful Grant Proposals: An Information Synthesis.” Academic Medicine 90, no. 12: 1720–1725. 26200582 10.1097/ACM.0000000000000811

[jgc470259-bib-0018] Zierhut, H. , S. Betterman , M. Kocher , H. Tsegai , and R. Mills . 2024. “The State of National Institute of Health Awards for Funding Genetic Counseling Research, Resources, and Training Over the Past Decade.” Journal of Genetic Counseling 33, no. 6: 1159–1175. 10.1002/jgc4.1850. 38264803 PMC11632580

