# Supplementary material for: Genetic Counselors as Research Leaders: A Pragmatic Pathway to Becoming a Federally Funded Principal Investigator in the United States
Source: J Genet Couns. 2026 Jul 17;35(4):e70259. doi: 10.1002/jgc4.70259 (PMC13379507; doi:10.1002/jgc4.70259)
Supplement: Supplementary file 1 — Appendix S1: Interview Guide for Non‐GC Researchers. [file JGC4-35-0-s001.pdf]

**Supplement 1: Interview Guide For Non-GC Researchers**

**Starting Question**

1) Your research study [study name] was identified in a review of NIH Reporter as a study involving genetic counseling. Could you give me your quick 'elevator spiel' of the study?

**Involvement of GCs as PIs**

2a) Could you tell me a bit about the process of how you identify members of the research team like co-investigators or others in leadership roles?

- Who all is involved in helping make that decision?

2b) You mentioned that genetic counseling was part of the study-- was that work conducted by certified genetic counselors?

- What roles did genetic counselors have in your study?
- *If GC was a PI:* What were the reasons that a genetic counselor was chosen to be a PI?
    - What skills do you think the genetic counselors brought as PIs?
- *If GC was not a PI but was on team*: What roles did those genetic counselors have on the team?
    - See question 3

2c) **Have you been a part of any previous research projects where genetic counselors were PIs?**

- If so, was this NIH-funded?
- What was the topic/focus of the research? Study design (clinical trial, observational)?
- What skills do you think the genetic counselors brought as PIs?
- Were there any skills that they did not have and needed to learn?
    - Does your institution or research group offer any resources for genetic counselors or other junior researchers to build these skills?

DO NOT USE WITHOUT AUTHOR PERMISSION

**Reasons not a PI**

3a) **Did you consider having a genetic counselor as a PI or Co-PI?**

- *If they considered having a GC as a PI, what prevented that from happening:*
    - What were the reasons you considered having a genetic counselor as a PI?
    - What ultimately led you to go another direction?
    - Did you not know any genetic counselors at your institution or elsewhere to reach out to?
    - *If GC was not interested:* What were the reasons the genetic counselor gave you about why they decided not to be a PI?
        - Didn't have time? Didn't feel they had the research experience they needed?

- *If they did NOT consider having a GC as a PI:*
    - What influenced that decision?
    - See question 3

3b) **Some genetic counselors may have limited training related to NIH-funded research and managing large grants. How might GC's training impact their ability to be PIs?**

- Could you describe the skillset that you think genetic counselors would need in order to be supported as a PI or co-PI?
    - Knowledge about  NIH research process and rules? Regulatory guidelines? Statistics? Content knowledge (medical or basic science)?
    - Skills  Grant writing? Previous publications? Experience designing complex trials? Consenting and interacting with participants?
- Could you describe what skillset you think genetic counselors already have that would be beneficial to them as PIs?

3c) **Other potential probes into the reasons**

- Do you think that this just wasn't something people thought about-- having a genetic counselor as a PI?
- Do you think people were aware that genetic counselors can have research roles?
    - What do you think is the general awareness among your research colleagues (e.g. PhDs, MDs) about there being research genetic counselors?
- How do you think credentials (having a PhD versus a Master's) factor into the decision?
    - *If they prefer PhD:* Do you think having someone with a Master's as PI may affect chances the grant will be funded?
- Is there a certain type of NIH-funded research (e.g. clinical trials, observational study) where you think genetic counselors would be particularly well-suited as PIs?
    - What about research topic? Are there certain topics where you think genetic counselors would be able to step into that role more easily than other research topics?

3d) **What would make you more likely to include a genetic counselor as part of your research leadership team on a future genetic counseling research project?**

3e) **Is there any other type of training or skill development that you can think of that would be helpful to genetic counselors who are hoping to take on PI roles?**

- What might you do to support or encourage genetic counselors to lead or co-lead research studies similar to yours?

DO NOT USE WITHOUT AUTHOR PERMISSION
