# Supplementary material for: Genetic Counselors as Research Leaders: A Pragmatic Pathway to Becoming a Federally Funded Principal Investigator in the United States
Source: J Genet Couns. 2026 Jul 17;35(4):e70259. doi: 10.1002/jgc4.70259 (PMC13379507; doi:10.1002/jgc4.70259)
Supplement: Supplementary file 2 — Appendix S2: Interview Guide for GC Researchers. [file JGC4-35-0-s002.pdf]

Supplement 2: Interview Guide for GC Researchers

Starting Question

1) Your research study [study name] was identified either in a review of NIH Reporter or self-identified as being a PI on a NIH or equivalent type of study involving genetic counseling. Could you give me your quick 'elevator spiel' of the study?

GCs as PIs – training facilitators, barriers, etc.

2a) Could you tell me a bit about the process of how you became a leader (or Co-I) of the research team?

2b) On this specific grant and over the course of your career, who was involved in helping you to become the PI or Co-I?

*** 2c/d only for PIs***
2c) What were the biggest facilitators of your success in obtaining funding for genetic counseling research?

2d) What were your biggest barriers to your success in obtaining funding for genetic counseling research?

2e) Have you been a part of any previous federal research projects where genetic counselors where you were a Co-I?

- What was the topic/focus of the research? Study design (clinical trial, observational)?
- What skills do you think you brought to the study as a Co-I?
- Were there any skills that you did not have and needed to learn?
    - Does your institution or research group offer any resources for genetic counselors or other junior researchers to build these skills?

DO NOT USE WITHOUT AUTHOR PERMISSION

Genetic Counseling Research Training & Skills

3a) What additional training did you get, if any, to help prepare you to do genetic counseling research?

3b) How might GC's training impact their ability to be PIs?

- Prompt #1: How do you think credentials (having a PhD versus a Master's) factor into the decision to apply for federally funded?
    - *If they had a MS:* Do you think having a Master's as PI affected your chances the grant will be funded, if so why or why not?
    - *If they had a PhD*: Do you think having a Doctoral degree as PI may affected your chances the grant will be funded? why or why not.
- Prompt #2: Is there a certain type of NIH-funded research (e.g. clinical trials, observational study) where you think genetic counselors would be particularly well-suited as PIs?

3c) What do you think would make it more likely for a genetic counselor to be the PI of a grant on a future genetic counseling research project?

Genetic Counseling Grant Writing Training & Skills

4a) Genetic counselors have varied training related to NIH-funded research and managing large grants. What roles did you have in your grant preparation? and on the study overall?

4b) And was there anything unique that you did for writing and securing grants?

- *If GC was the only PI:* For what reasons did you think that having a genetic counselor be the PI was important?
- *If GC was a multiple PI*: What roles did you versus the other PI have on the grant leadership?

Overall Training & Skills

5a) Is there any other type of training or skill development that you can think of that would be helpful to genetic counselors who are hoping to take on PI roles for federally funded studies?

- What might you do to support or encourage genetic counselors to lead or co-lead research studies similar to yours?

DO NOT USE WITHOUT AUTHOR PERMISSION
